# A large pruritic plaque in a patient with 2 kidney transplants

**DOI:** 10.1016/j.jdcr.2024.12.028

**Published:** 2025-01-10

**Authors:** Chiel F. Ebbelaar, Sadhanna Badeloe, Anne M.R. Schrader, Elsemieke I. Plasmeijer

**Affiliations:** a Department of Dermatology, Leiden University Medical Center, Leiden, The Netherlands; b Division of Laboratories, Pharmacy and Biomedical Genetics, Department of Pathology, University Medical Center Utrecht, Utrecht, The Netherlands; c Department of Pathology, Leiden University Medical Center, Leiden, The Netherlands; d Department of Dermatology, Netherlands Cancer Institute, Amsterdam, The Netherlands

**Keywords:** cornoid lamellae, giant porokeratosis, immunosuppression, kidney transplantation, skin cancer, topical simvastatin

## Case presentation

**Fig 1:**
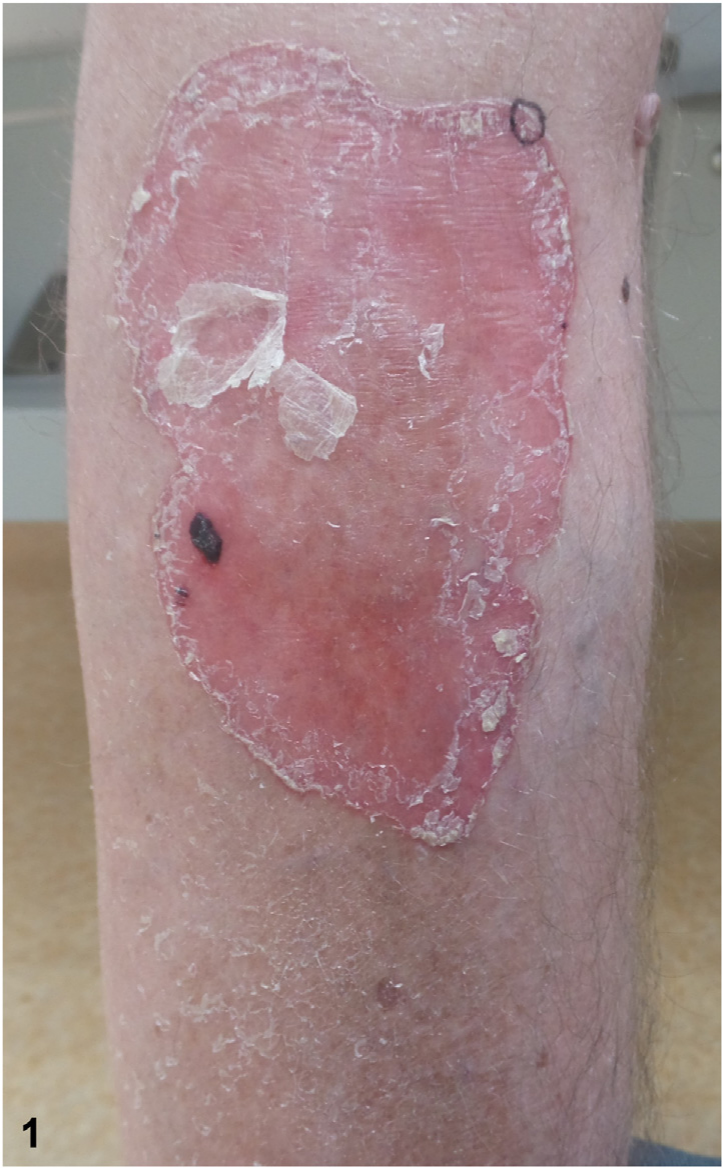

**Fig 2:**
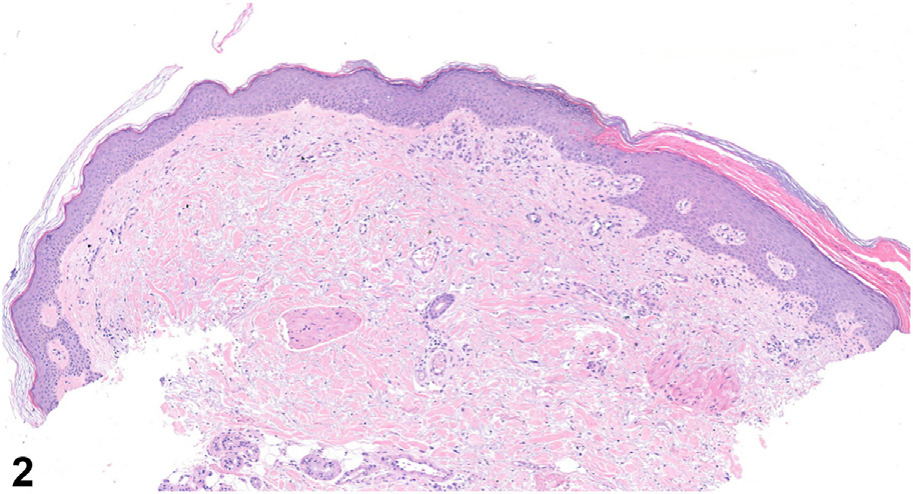

A 58-year-old man with a history of 2 kidney transplants presented with a progressively expanding, pruritic, and slightly painful erythematous plaque (23 × 13 cm) on his right lower leg that had developed over 3 years (Fig 1). His dermatological history included actinic keratosis, squamous cell carcinoma, and basal cell carcinoma. He was currently on immunosuppressive therapy with prednisolone (7.5 mg daily) and ciclosporin (75 mg twice daily), as well as levodopa/carbidopa for Parkinson disease and pravastatin for hypercholesterolemia. Previous treatments with topical corticosteroids, emollients, and 5-fluorouracil had been unsuccessful. A potassium hydroxide preparation and skin biopsy were performed to aid diagnosis (Fig 2).

**Question 1: What is the patient’s diagnosis?**

A. Giant porokeratosis
B. Dermatophytosis
C. Actinic keratosis
D. Squamous cell carcinoma
E. Psoriasis

**Answers:**

A. Giant porokeratosis – Correct. The skin biopsy revealed characteristic cornoid lamellae, a hallmark of porokeratosis, at the squamous collarette border (Fig 2). The cornoid lamella consists of a column of parakeratosis with underlying dyskeratotic keratinocytes and a reduction of the granular layer, confirming the diagnosis of porokeratosis. Giant porokeratosis is a rare subtype characterized by solitary plaques that can cover extensive skin areas, often associated with ultraviolet radiation exposure and immunosuppression, particularly in individuals who have undergone solid organ or bone marrow transplants. Similar to other forms of porokeratosis, the presence of cornoid lamellae distinguishes it from other conditions.
B. Dermatophytosis – Incorrect. Both the potassium hydroxide preparation and polymerase chain reaction were negative for dermatophytosis, ruling out this diagnosis.
C. Actinic keratosis – Incorrect. Actinic keratosis typically appears on sun-exposed areas and is histologically characterized by cytological atypia of keratinocytes, which is not present in this case. Actinic keratosis may mimic cornoid lamellae due to the formation of columns of parakeratosis overlying the atypical keratinocytes, but it lacks the characteristic dyskeratosis of true cornoid lamellae.
D. Squamous cell carcinoma – Incorrect. Squamous cell carcinoma is histologically characterized by a proliferation of squamous epithelium with cytological atypia and infiltrative growth. While giant porokeratosis may progress to squamous cell carcinoma in some cases, the biopsy showed none of these features.^1^
E. Psoriasis – Incorrect. The characteristic histological features of psoriasis, including regular acanthosis with confluent parakeratosis, often accompanied by neutrophils in the corneal layer, were absent in the biopsy. Also, psoriasis lacks cornoid lamellae and does not display a collarette border clinically.^1^

**Fig 3:**
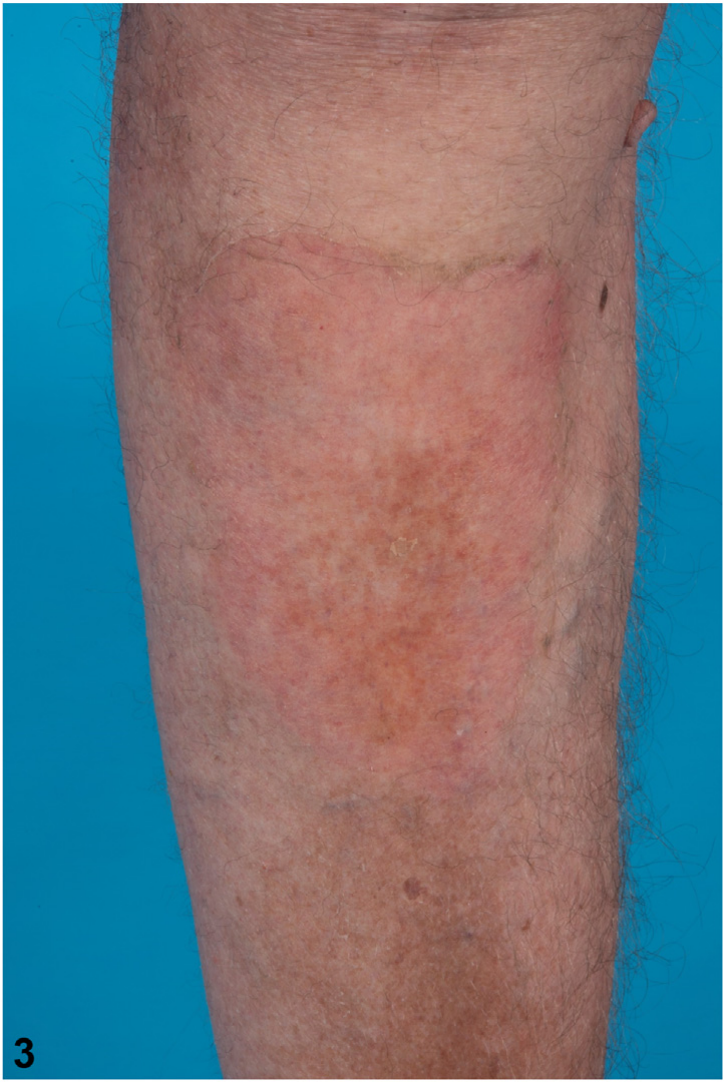
D. Topical tretinoin – Incorrect. While topical tretinoin has been reported to have mixed efficacy in treating porokeratosis, clinical results are often disappointing.^4^
E. Photodynamic therapy – Incorrect. Photodynamic therapy has primarily been investigated for disseminated superficial actinic porokeratosis, showing limited effectiveness and a potentially unfavorable safety profile.^4^

**Question 2: Which treatment option was likely used to achieve the results shown in** Fig 3 **?**

A. Oral statin therapy
B. Topical 5-fluorouracil cream
C. Topical simvastatin cream
D. Topical tretinoin
E. Photodynamic therapy

**Answers:**

A. Oral statin therapy – Incorrect. Oral statins have not been demonstrated to be effective in treating porokeratosis. While the exact reason for their lack of efficacy is not fully understood, it is likely due to low skin bioavailability, as oral statins undergo extensive first-pass metabolism in the liver and have limited penetration into the skin. Additionally, the patient had been on pravastatin for years prior to the development of this lesion, further supporting the ineffectiveness of oral statins in this context.
B. Topical 5-fluorouracil cream – Incorrect. Traditional therapies, including topical 5-fluorouracil, often fail to provide significant or lasting improvements in giant porokeratosis, as evidenced in this case.
C. Topical simvastatin cream – Correct. Topical statins have shown effectiveness in treating various subtypes of porokeratosis, although their efficacy for giant porokeratosis is not extensively documented.^2^^,^^3^ The patient was started on a trial with 2% simvastatin cream, applied twice daily. After 2 weeks, he reported marked improvement in the lesion and significant reduction in pruritus, with no side effects. At a follow-up visit 3 months later, the lesion had nearly resolved, leaving only postinflammatory hyperpigmentation and slight erythema (Fig 3).

**Question 3: What is the primary treatment mechanism of simvastatin in porokeratosis?**

A. Normalizing keratinocyte proliferation
B. Stimulates immune response in keratinocytes
C. Increases epidermal turnover
D. Enhances collagen synthesis
E. Induces apoptosis in keratinocytes

**Answers:**

A. Normalizing keratinocyte proliferation – Correct. Recent studies have linked porokeratosis with mutations in genes associated with the mevalonate pathway (m*evalonate kinase*, *phosphomevalonate kinase*, *mevalonate diphosphate decarboxylase*, and *farnesyl diphosphate synthase*). The therapeutic action of topical simvastatin in porokeratosis likely involves inhibition of 3-hydroxy-3-methylglutaryl-coenzyme A reductase reductase within this pathway, thereby preventing the accumulation of toxic metabolic intermediates and normalizing keratinocyte proliferation and differentiation.^5^
B. Stimulates immune response in keratinocytes – Incorrect. Simvastatin primarily modulates lipid pathways rather than activating the immune response.
C. Increases epidermal turnover – Incorrect. Simvastatin is more effective at reducing keratinocyte proliferation than increasing epidermal turnover.
D. Enhances collagen synthesis – Incorrect. The primary effect of simvastatin is on keratinocytes, with no direct influence on collagen synthesis.
E. Induces apoptosis in keratinocytes – Incorrect. Simvastatin does not induce apoptosis in keratinocytes. Instead, it works by inhibiting 3-hydroxy-3-methylglutaryl-coenzyme A reductase reductase, which reduces toxic intermediates in the mevalonate pathway, thus normalizing keratinocyte proliferation and differentiation rather than promoting cell death.

## Conflicts of interest

None disclosed.
